# Severe Acute Respiratory Syndrome Coronavirus 2 Did Not Substantially Impact Injury Patterns or Performance of Players in the National Basketball Association From 2016 to 2021

**DOI:** 10.1016/j.asmr.2023.100841

**Published:** 2023-12-21

**Authors:** Sachin Allahabadi, Anoop R. Galivanche, Nathan Coss, Norbu Tenzing, Andrew P. Gatto, Jerome C. Murray, Sameer Allahabadi, Nirav K. Pandya

**Affiliations:** a Department of Orthopaedic Surgery, University of California San Francisco, San Francisco, California, U.S.A.; b School of Medicine, University of California San Francisco, San Francisco, California, U.S.A.; c College of Osteopathic Medicine, Touro University California, Vallejo, California, U.S.A.; d Department of Orthopaedic Surgery, University of Minnesota, Minneapolis, Minnesota, U.S.A.; e Baylor Scott & White Institute for Rehabilitation, Baylor University Medical Center, Dallas, Texas, U.S.A.

## Abstract

**Purpose:**

To perform a descriptive epidemiologic analysis of National Basketball Association (NBA) injuries from 2016 to 2021, to evaluate the impact of severe acute respiratory syndrome coronavirus 2 (SARS-CoV-2) (coronavirus disease 2019, or COVID-19) on injury patterns and performance statistics, and to determine the effect of infection with SARS-CoV-2 on individual performance statistics.

**Methods:**

Injury epidemiology in the NBA from the 2016 to 2021 seasons was collected using a comprehensive online search. Injuries and time missed were categorized by injury location and type. Player positions and timing of injury were recorded. Performance statistics were collected including traditional game statistics and Second Spectrum (speed, distance) statistics. Comparisons were made over seasons and comparing the pre-COVID-19 pandemic seasons to the pandemic era seasons. Players diagnosed with COVID-19 were analyzed for changes in performance in the short or long term.

**Results:**

Of the 3,040 injuries captured, 1,880 (61.84%) were in the lower extremity. Guards (77.44%) and forwards (75.88%) had a greater proportion of soft-tissue injuries (*P* < .001) than centers. Guards had the highest proportion of groin (3.27%, *P* = .001) and hamstring (6.21%, *P* < .001) injuries. Despite minor differences on a per-season basis, there were no differences in injury patterns identified between pre-COVID-19 and COVID-19 eras. Of players diagnosed with COVID-19 during the NBA Bubble, there were no detriments in short- or long-term performance identified, including traditional game statistics and speed and distance traveled.

**Conclusions:**

In the NBA seasons from 2016 to 2021, most injuries were to the lower extremity. The SARS-CoV-2 pandemic did not substantially impact injury patterns in the NBA, including locations of injury and type of injury (bony or soft tissue). Furthermore, infection with SARS-CoV-2 does not appear to have a significant impact on performance in basketball-specific or speed and distance measures.

**Level of Evidence:**

Level IV, prognostic case series.

Injury analysis in professional athletes has been a topic of research and interest, as these athletes represent the highest level of activity and extremes of physical demands on their bodies. Epidemiologic analyses of injuries in these elite athletes are helpful to understand risks and impacts of injuries on performance, and, with this information, it is possible that changes in modifiable risks can be made to improve player safety. In the National Basketball Association (NBA), which is considered by many to be the highest standard for basketball, analyses of the effects of musculoskeletal injuries,^1^ surgical procedures,^2^ game load and fatigue,^3^^,^^4^ travel,^5^^,^^6^ and sleep^7^^,^^8^ on game play, performance, and career longevity have been performed.

Severe acute respiratory syndrome coronavirus 2 (SARS-CoV-2; also referred to as coronavirus disease 2019 [COVID-19]), a respiratory airborne virus, began rapidly spreading throughout the world at the end of 2019 and was declared a pandemic by the World Health Organization on March 11, 2020.^9^ Immediately after, the NBA temporarily suspended all game play beginning March 12, 2020.^10^ In June of 2020, the NBA Bubble^11^ was introduced after a several-month break in practice and play, with a unique format^12^ to isolate and protect NBA players from high-risk exposures to COVID-19. Because of this, the NBA season in 2019 to 2020 ended later and the time between the end of the 2019 to 2020 and 2020 to 2021 season was shorter than typical, affording players and especially those in the playoffs less time for recovery.

Although many specific musculoskeletal injuries have been evaluated in how they affect expected performance in elite basketball players, less has been studied on medical conditions and in particular on viruses and respiratory illness. Specifically, little is known about how COVID-19 impacts individual basketball performance. Many after being infected with COVID-19 report ongoing fatigue and limited ability to partake in aerobic exercise.^13^, ^14^, ^15^ Furthermore, with complications such as long COVID-19, it is possible that COVID-19 may have effects beyond the acute infectious and symptomatic period.^13^^,^^15^ Although some studies have evaluated the impacts of the pandemic on injury rates,^16^^,^^17^ few studies have examined the direct impacts on injury epidemiology and player performance.

The purposes of this study were to perform a descriptive epidemiologic analysis of NBA injuries from 2016 to 2021, to evaluate the impact of SARS-CoV-2 (COVID-19) on injury patterns and performance statistics, and to determine the effect of infection with SARS-CoV-2 on individual performance statistics. The authors hypothesized that lower-extremity injuries would be the most common and that SARS-CoV-2 would not affect injury patterns or have impacts on individual player performance.

## Methods

### Injury Epidemiology Study Data

The study was performed using publicly available online data through a comprehensive search.

Using the prosportstransactions.com archive as previous studies have done,^4^^,^^18^^,^^19^ players from the 2016 to 2017, 2017 to 2018, 2018 to 2019, 2019 to 2020, and 2020 to 2021 NBA seasons were identified having transactions related to injuries. For each injury, the following information was gathered: team, player name, injury/transaction type, date of birth, player position (guard, forward, center), injury setting (game, practice, unknown), injury timing (offseason, preseason, season, postseason), injury general location (upper extremity, lower extremity, spine/core, other), injury specific location specifying joint or location involved, and injury/transaction classification (soft tissue, bony, health and safety/COVID-19, other/non-musculoskeletal). If a player underwent surgery, this was noted as well.

### Performance Statistics Study Data

Players identified to have contracted SARS-CoV-2 during the 2019 to 2020 season were identified and confirmed with multiple online news resources. For these players, performance statistics were documented for the following time frames: the 2018 to 2020 season until the player was identified to have SARS-CoV-2, 2 weeks of play after returning from SARS-CoV-2, 1 month of play after returning, and the following 2020 to 2021 season after contracted SARS-CoV-2. Performance data analyzed were classified as game statistics or Second Spectrum statistics and are included in Table 1. Game statistics data were obtained via BasketballReference.com. Second Spectrum statistics were obtained using NBA.com. Second Spectrum (secondspectrum.com) is the Official Tracking Provider for the NBA and began tracking NBA players in the 2017-2018 season using an optical camera tracking system to obtain unique player statistics such as speed and distance.^20^

**Table 1:** Performance Data Including Game Statistics and Second Spectrum Statistics Analyzed in Players Identified to Have Had SARS-CoV-2 in the 2019-2020 NBA Season

Game Statistics (Abbreviation)	Second Spectrum Statistics (Abbreviation) [units]
Games (G)	Distance Feet, Total (Dist. Feet) [feet]
Games started (GS)	Distance Miles, Total (Dist. Miles) [miles]
Minutes played (MIN)	Distance Miles, Offensive (Dist. Miles Off) [miles]
Field goals (FG)	Distance Miles, Defensive (Dist. Miles Def) [miles]
Field goal attempts (FGA)	Speed [miles per hour]
Field goal % (FG%)	Speed, Offensive (Speed Off) [miles per hour]
3-pointers (3P)	Speed, Defensive (Speed Def) [miles per hour]
3-point attempts (3PA)	
3-point % (3P%)	
2-pointers (2P)	
2-point attempts (2PA)	
2-point % (2P%)	
Effective field goal % (eFG%)	
Free throws (FT)	
Free throw attempts (FTA)	
Free throw % (FT%)	
Offensive rebounds (ORB)	
Defensive rebounds (DRB)	
Total rebounds (TRB)	
Assists (AST)	
Steals (STL)	
Blocks (BLK)	
Turnovers (TOV)	
Personal fouls (PF)	
Points (PTS)	

NBA, National Basketball Association; SARS-CoV-2, severe acute respiratory syndrome coronavirus 2.

### Statistical Analyses

Epidemiologic analyses were first performed. Injury data were characterized for descriptive analyses. Proportions of injuries by general region, specific location, and type (soft tissue, bony, miscellaneous) were compared over time using χ^2^ tests. The 2016 to 2017, 2017 to 2018, 2018 to 2019, and 2019 to 2020 (pre-NBA Bubble) injuries were then considered to be in the pre-COVID-19 era and compared with the 2019 to 2020 (during NBA Bubble) and 2020 to 2021 season using χ^2^ tests. In addition to the temporal analyses, general regions, specific locations, and types of injury also were characterized by player position and compared using χ^2^ tests. Injuries per games played in each season were grossly estimated by dividing the number of injuries in the season by the average number of regular season games played in that season and rates were compared using Poisson method.

Comparative analyses of performance data for players diagnosed with COVID-19 were then performed. These players were compared with themselves from the season before COVID-19 (2018-2019), 2019 to 2020 before being diagnosed with COVID-19, 2019 to 2020 after being diagnosed with COVID-19, and the following season 2020 to 2021. Within the 2019-2020 season, if they had games still to play, they were compared at return approximately 2 weeks’ post-COVID-19, 1 month after being diagnosed with COVID-19, and until the end of the 2019-2020 season if they continued to play 1 month onward. Percentage-based statistics (field goal percentage, 3-point percentage, 2-point percentage, effective field goal percentage, free throw percentage) and other traditional in-game statistics (evaluated on a per-game basis) were compared.

Missingness of data was assessed per variable and was primarily due to player absence in that sampling interval. To account for repeated measures by player and missing data, time-varying generalized linear models with random intercepts for each player were constructed. Count data were modeled using Poisson link functions. Continuous data were modeled using Gaussian link functions. Null models without a time fixed effect but including nested player effects were also constructed. χ^2^ tests comparing each time varying model to its corresponding null model were used to determine whether the addition of a time variable resulted in a statistically significant reduction in the residual sum of squares. When performance metrics were found to have a statistically significant time-varying component, post-hoc tests were performed using pairwise Fisher exact tests, with the family-wise error rate controlled using Bonferroni correction.

Statistical analyses were performed using R (R Foundation for Statistical Computing, Vienna, Austria) with computing packages dplyr (dplyr: A Grammar of Data Manipulation. R package version 0.8.5), magrittr (magrittr: A Forward-Pipe Operator for R. R package version 1.5), lme4 (lme4: Linear Mixed-Effects Models using 'Eigen' and S4. R package version 1.1-32), and lmerTest (lmerTest: Tests in Linear Mixed Effects Models. R package version 3.1-3).

## Results

### Epidemiologic Analyses: Injuries by Location and Type

There were 3,040 injuries captured in the epidemiologic analysis (Table 2). Injuries to the lower extremity (1,880, 61.84%) represented the majority. Specific injury locations and their overall proportions are demonstrated in Table 2. In terms of injury types, 2,160 (71.1%) were primarily soft-tissue injuries, 133 (4.4%) were primarily bony injuries, and 576 (18.9%) were invocations of health/safety protocols or categorized as miscellaneous (Table 2).

**Table 2:** NBA General Injury Characteristics From 2016-2021, Overall

	Total		Age		Underwent Surgery	
	Number	Percentage	Mean	Std. Dev.	Number	Percentage
General location characteristics						
All	3,040	100.0%	27.2	4.3	171	5.63%
Lower extremity	1,880	61.8%	27.0	4.2	114	6.1%
Upper extremity	333	11.0%	27.0	4.3	43	12.9%
Spine/core	230	7.6%	27.8	4.6	6	2.6%
Other	597	19.6%	27.6	4.7	8	1.3%
Specific injury region characteristics						
Head	120	3.9%	25.7	3.5	4	3.3%
Neck	26	0.9%	28.5	4.9	0	0.0%
Chest	13	0.4%	29.8	6.2	1	7.7%
Shoulder	102	3.4%	26.6	4.2	18	17.6%
Elbow/forearm	38	1.3%	26.6	4.4	1	2.6%
Hand/wrist	185	6.1%	27.4	4.2	26	14.1%
Abdomen	19	0.6%	25.3	3.5	2	10.5%
Groin	71	2.3%	27.9	4.3	4	5.6%
Hamstring	124	4.1%	28.4	4.0	1	0.8%
Hip	118	3.9%	27.4	4.3	1	0.8%
Knee	556	18.3%	27.0	3.9	71	12.8%
Lower back	172	5.7%	27.8	4.4	3	1.7%
Foot/ankle	703	23.1%	26.3	4.2	27	3.8%
Infection	298	9.8%	27.1	4.6	0	0.0%
Miscellaneous	179	5.9%	29.8	4.7	4	2.2%
Other lower extremity	308	10.1%	27.7	4.4	11	3.6%
Other upper extremity	8	0.3%	24.0	3.4	0	0.0%
Characteristics of injury types						
Soft tissue	2,160	71.1%	27.1	4.2	138	6.4%
Bony	133	4.4%	26.8	4.2	27	20.3%
Health and safety/COVID-19 and Miscellaneous	576	18.9%	27.6	4.7	6	1.0%

COVID-19, coronavirus 2019; NBA, National Basketball Association; SARS-CoV-2, severe acute respiratory syndrome coronavirus 2.

### Epidemiologic Analyses: Injuries by Year

Over time, there were significant differences in the distribution of injuries by general region (lower extremity, upper extremity, spine/core, other) over the years tracked; however, none of these were significant when comparing the COVID-19 era with the pre-COVID-19 era (Table 3). The proportion of infection-related transactions was significantly different between the pre-COVID-19 and COVID-19 era (8.25% in the pre-COVID-19 era compared with 16.73% in the COVID-19 era, *P* < .001). There were no differences in the distribution of musculoskeletal injury types (soft tissue or bony) before and during the COVID-19 era (Table 3). When we compared grossly estimated rates of injuries, the 2016 to 2017 season had a significantly greater rate than all other seasons (*P* < .01). The 2018 to 2019 season had a significantly lower rate of injuries than all other seasons (*P* < .05). There was no difference in rates comparing the 2017 to 2018 with either the 2019 to 2020 (*P* = .49) or 2020 to 2021 (*P* = .79) seasons. There was no difference in injury rate between the 2019 to 2020 and 2020 to 2021 seasons (*P* = .67).

**Table 3:** NBA Injury Characteristics From 2016-2021, by Year

	Total		2016-2017		2017-2018		2018-2019		2019-2020		2020-2021		Across All Years	COVID-19
	Number	Percentage	Number	Percentage	Number	Percentage	Number	Percentage	Number	Percentage	Number	Percentage	*P* Value∗	*P* Value∗
All	3,040	100.0%	810	26.64%	643	21.15%	542	17.83%	489	16.09%	556	18.29%	–	–
General regions of injury over time														
Lower extremity	1,880	61.8%	463	57.16%	394	61.28%	368	67.90%	301	61.55%	354	63.67%	.002	.327
Upper extremity	333	11.0%	95	11.73%	83	12.91%	49	9.04%	61	12.47%	45	8.09%	.028	.017
Spine/core	230	7.6%	37	4.57%	52	8.09%	55	10.15%	49	10.02%	37	6.65%	<.001	.369
Other	597	19.6%	215	26.54%	114	17.73%	70	12.92%	78	15.95%	120	21.58%	<.001	.202
Specific regions of injury over time														
Head	120	3.95%	31	3.83%	28	4.35%	18	3.32%	24	4.91%	19	3.42%	.649	.478
Neck	26	0.86%	1	0.12%	5	0.78%	7	1.29%	8	1.64%	5	0.90%	.042	.901
Chest	13	0.43%	3	0.37%	6	0.93%	2	0.37%	2	0.41%	0	0.00%	.174	.087
Shoulder	102	3.36%	27	3.33%	28	4.35%	12	2.21%	23	4.70%	12	2.16%	.053	.083
Elbow/forearm	38	1.25%	9	1.11%	10	1.56%	9	1.66%	7	1.43%	3	0.54%	.440	.095
Hand/wrist	185	6.09%	56	6.91%	42	6.53%	26	4.80%	31	6.34%	30	5.40%	.511	.452
Abdomen	19	0.63%	2	0.25%	5	0.78%	4	0.74%	6	1.23%	2	0.36%	.223	.380
Groin	71	2.34%	18	2.22%	17	2.64%	10	1.85%	10	2.04%	16	2.88%	.776	.349
Hamstring	124	4.08%	36	4.44%	20	3.11%	24	4.43%	29	5.93%	15	2.70%	.062	.069
Hip	118	3.88%	30	3.70%	21	3.27%	19	3.51%	25	5.11%	23	4.14%	.556	.730
Knee	556	18.29%	140	17.28%	119	18.51%	112	20.66%	81	16.56%	104	18.71%	.455	.779
Lower back	172	5.66%	31	3.83%	36	5.60%	42	7.75%	33	6.75%	30	5.40%	.030	.767
Foot/ankle	703	23.13%	171	21.11%	152	23.64%	143	26.38%	105	21.47%	132	23.74%	.195	.703
Infection	298	9.80%	64	7.90%	42	6.53%	47	8.67%	52	10.63%	93	16.73%	<.001	<.001
Miscellaneous	179	5.89%	120	14.81%	44	6.84%	5	0.92%	2	0.41%	8	1.44%	<.001	<.001
Other lower extremity	308	10.13%	69	8.52%	64	9.95%	60	11.07%	51	10.43%	64	11.51%	.397	.233
Other upper extremity	8	0.26%	2	0.25%	4	0.62%	2	0.37%	0	0.00%	0	0.00%	.188	.180
Injury types over time														
Soft tissue	2,298	75.59%	553	68.27%	490	76.21%	456	84.13%	386	78.94%	413	74.28%	<.001	.426
Bony	160	5.26%	47	5.80%	45	7.00%	18	3.32%	27	5.52%	23	4.14%	.042	.188
Health and safety/COVID-19 and miscellaneous	582	19.14%	210	25.93%	108	16.80%	68	12.55%	76	15.54%	120	21.58%	<.001	.106

COVID-19, coronavirus disease 2019; NBA, National Basketball Association; SARS-CoV-2, severe acute respiratory syndrome coronavirus 2.

∗ Bold *P* values significant at *P* < .002 (Bonferroni correction).

### Epidemiologic Analyses: Injuries by Season Timing

There was a significant difference by season timing in bony injuries (highest, 33.33%, in the off season; lowest, 4.82%, during the season; *P* < .001). There were no differences in season timing in the proportion of injuries by general or specific location (Appendix Table 1, available at www.arthroscopyjournal.org).

### Epidemiologic Analyses: Injuries by Player Position

Guards (77.44%) and forwards (75.88%) had a greater proportion of soft-tissue injuries than centers (67.91%) (*P* < .001). Guards had the greatest proportion of groin injuries (3.27%, *P* = .001) and hamstring injuries (6.21%, *P* < .001). Additional evaluations by player position are noted in Appendix Table 2, available at www.arthroscopyjournal.org.

### Performance Statistics

Thirty-two players diagnosed with COVID-19 during the NBA Bubble were included in COVID-19–specific performance analyses.

### Performance Statistics: Per-Game Statistics

Per-game statistics for players eventually diagnosed with COVID-19 during the NBA Bubble by season are demonstrated in Table 4. Games played and games started decreased significantly over the 3 seasons sampled. There were no significant differences across seasons in any of the in-game performance statistics evaluated when normalized on a per-game basis (Table 4).

**Table 4:** Per Game Statistics Over Time for Players Diagnosed With COVID

	2018-2019		2019-2020		2020-2021		Pre- vs Post-COVID-19
	Mean	Std. Dev.	Mean	Std. Dev.	Mean	Std. Dev.	*P* Value∗
Games (G)	61.40	22.00	54.90	14.00	49.20	22.10	<.001
Games started (GS)	39.70	31.50	37.70	23.90	36.80	24.80	<.001
Per-game statistics							
Minutes played (MIN)	23.60	8.17	26.10	7.20	25.30	9.12	.151
Field goals (FG)	4.47	2.22	4.80	2.23	4.64	2.61	.465
Field goal attempts (FGA)	9.24	4.90	10.10	4.88	9.46	5.49	.276
3-pointers (3P)	1.07	0.87	1.16	0.91	1.27	1.04	.445
3-point attempts (3PA)	3.00	2.22	3.42	2.43	3.34	2.63	.556
2-pointers (2P)	3.39	1.86	2.93	2.82	3.38	2.30	.667
2-point attempts (2PA)	6.23	3.47	5.57	5.49	6.12	4.18	.612
Free throws (FT)	2.08	1.37	2.16	1.39	2.15	1.53	.555
Free throw attempts (FTA)	2.75	1.74	2.81	1.83	2.82	1.89	.759
Offensive rebounds (ORB)	1.24	0.89	1.32	0.83	1.16	0.88	.187
Defensive rebounds (DRB)	3.91	2.43	4.32	2.01	4.29	2.43	.289
Total rebounds (TRB)	5.16	3.17	5.67	2.74	5.45	3.11	.329
Assists (AST)	2.68	2.41	3.11	2.41	3.06	2.78	.263
Steals (STL)	0.76	0.47	0.77	0.44	0.72	0.39	.450
Blocks (BLK)	0.60	0.45	0.60	0.44	0.62	0.56	.885
Turnovers (TOV)	1.54	0.98	1.70	0.97	1.57	1.04	.115
Personal fouls (PF)	2.16	0.64	2.31	0.61	1.93	0.67	.003
Points (PTS)	12.10	6.19	12.90	5.99	12.70	7.12	.523

COVID-19, coronavirus disease 2019.

∗ Bold *P* values are significant at *P* < .002 (Bonferroni correction).

### Performance Statistics: Percentage-Based Game Statistics

Percentage-based game statistics were compared across every sampling interval for players eventually diagnosed with COVID-19 during the NBA Bubble (Table 5). There were no significant differences across time points in effective field goal percentage, field goal percentage, 3-point percentage, 2-point percentage, or free-throw percentage.

**Table 5:** Percentage-Based Game Statistics Over Time in Players Diagnoses With COVID-19

	2018-2019		2019-2020		>2 Weeks Post-COVID-19		1-Month Post-COVID-19		>1-Month Post-COVID-19		2020-2021		Pre- vs Post-COVID-19
	Mean	Std. Dev.	Mean	Std. Dev.	Mean	Std. Dev.	Mean	Std. Dev.	Mean	Std. Dev.	Mean	Std. Dev.	*P* Value∗
Field goal % (FG%)	49.4	7.12	48.2	9.22	46.6	12.3	45.9	11.1	46.8	10.5	52.7	14.2	.080
3-Point % (3P%)	34.2	6.51	30.6	9.38	35.3	20.9	35.0	21.9	30.5	13.7	35.2	17.0	.737
2-Point % (2P%)	70.9	86.6	52.9	4.82	N/A	N/A	N/A	N/A	N/A	N/A	56.7	11.2	.574
Effective field goal % (eFG%)	55.0	5.56	52.0	9.89	59.3	11.8	59.0	12.6	60.4	10.7	60.5	19.4	.034
Free throw % (FT%)	75.0	10.9	99.5	12.7	75.2	14.9	76.0	20.0	76.8	17.0	76.0	12.1	.603

COVID-19, coronavirus disease 2019; N/A, not available.

∗ Bold *P* values significant at *P* < .01

### Performance Statistics: Second Spectrum Statistics

Second Spectrum statistics were also compared across every sampling interval, as these statistics are collected on a per-game basis. There were no statistically significant differences in total distance, offensive distance, defensive distance, offensive speed, or defensive speed across the sampled time points in any player diagnosed with COVID (Table 6).

**Table 6:** Second Spectrum Performance Statistics in Players Diagnosed With COVID-19

	2018-2019		2019-2020		>2 Weeks Post- COVID-19		1-Month Post-COVID-19		>1-Month Post- COVID-19		2020-2021		*P* Value
	Mean	Std. Dev.	Mean	Std. Dev.	Mean	Std. Dev.	Mean	Std. Dev.	Mean	Std. Dev.	Mean	Std. Dev.	
Distance miles, total (Dist. Miles) [miles]	1.80	0.56	1.90	0.59	1.90	0.60	1.88	0.58	1.89	0.49	1.84	0.61	.906
Distance miles, offensive (Dist. Miles Off) [miles]	0.97	0.31	1.01	0.33	1.03	0.33	1.01	0.32	1.01	0.27	1.00	0.34	.956
Distance miles, defensive (Dist. Miles Def) [miles]	0.83	0.25	0.85	0.27	0.88	0.27	0.87	0.26	0.88	0.22	0.84	0.27	.975
Speed [miles per hour]	4.22	0.22	4.16	0.25	4.18	0.24	4.17	0.21	4.13	0.20	4.21	0.22	0.435
Speed, Offensive (Speed Off) [miles per hour]	4.60	0.31	4.50	0.27	4.55	0.31	4.51	0.24	4.50	0.25	4.58	0.37	.290
Speed, defensive (Speed Def) [miles per hour]	3.88	0.26	3.81	0.25	3.81	0.23	3.83	0.25	3.78	0.20	3.90	0.26	.339

COVID-19, coronavirus disease 2019.

## Discussion

The most important finding of this study is that we found no major identified differences in injury patterns before the COVID-19 pandemic and during the COVID era, including location of injury or type of injury (bony or soft tissue). Diagnosis of SARS-CoV-2 did not impact traditional game performance or Second Spectrum statistics. In general, most musculoskeletal injuries occurred to the lower extremities. Overall, the findings support the authors’ hypotheses.

The data in the present study spanned 5 seasons, including 3 full seasons before the pandemic, the NBA Bubble Season, and the season following. This study not only characterized injuries and medical conditions by general region and type, but also by specific location. Overall, although there was some fluctuation in relative proportion of injuries over time from 2016 to 2021, these differences were not statistically significant. The COVID-19 pandemic, therefore, did not substantially impact injury patterns (proportions of injury types) in the NBA in the COVID-19 era. There have been mixed results on if the rates of injuries (ie, injuries per unit time or game played) increased in the condensed NBA seasons from the pandemic^16^^,^^17^; however, we did not find evidence supporting a greater rate of injuries in this study.

In addition to the pandemic not having a major impact on injury patterns, diagnosis of SARS-CoV-2 during the NBA Bubble did not impact game performance statistics. Specifically, in players diagnosed with COVID-19, there were no differences in offensive statistics (such as points, field goals, 3-pointers or 2-pointers, free throws, offensive rebounds) or defensive statistics (such as defensive rebounds, steals, blocks). These findings are in line with another study by Vaudreuil et al.^21^ In that study, the authors evaluated 20 players recovering from COVID-19 playing in the NBA Bubble and found that these athletes had a statistically reduced minutes per game (25.8 vs 28.7, *P* = .04) and statistically increased free throws per game (3.3 vs 2.8, *P* = .04) in the Bubble relative to career averages, but no other significant changes in performance.^21^ Our data do identify a reduction in games played and games started in the 2020 to 2021 season compared with previous seasons, recapitulating the abbreviated nature of the 2020 to 2021 season due to the COVID-19 pandemic. However, despite players participating in fewer games, their statistics on a per-game basis support that COVID-19 did not appear to have a meaningful impact on game play after return to sport.

Unique to this study is the incorporation of Second Spectrum statistics. There is limited literature examining these data in the NBA to date. This study provides a sample of 32 players for which Second Spectrum statistics are captured. Future studies may incorporate additional analyses of these unique datapoints. Although many patients report fatigue and difficulty with aerobic performance after COVID-19, in these elite level athletes no major changes were noted in distance (total, offensive, and defensive) or speed (total, offensive, and defensive) throughout the average game within the short- or long-term setting after diagnosis with COVID-19. This finding may support the notion that improved baseline physical activity and aerobic health may result in better long-term health outcomes after diagnosis with COVID-19.^22^, ^23^, ^24^

In terms of general epidemiology from 2016 to 2021, lower-extremity musculoskeletal injuries comprise the majority (∼62%) of injuries resulting in time missed in the NBA. These data are consistent with literature from the National Collegiate Athletic Association, which demonstrated approximately 60% of injuries to the lower extremity.^25^ A previous study from 1996 to 2002 in Women’s National Basketball Association and NBA additionally found a rate of ∼65% of injuries to the lower extremity,^26^ which is consistent with another study from 1988-2005^27^. Furthermore, a systematic review of orthopaedic and sports medicine-related injuries in the NBA and Women’s National Basketball Association through April 2020 found that 63.3% of available studies in these high-level basketball players were on lower extremity injuries.^1^ Therefore, in the context of previous literature, this study demonstrates a consistent proportion of lower extremity injuries over time.

Lower-extremity injuries also tended to be greater in guards (62.22%) and forwards (63.60%) relative to centers (55.47%), although not statistically significant. Guards also had the greatest proportion of hamstring (6.21%) and groin (3.27%) injuries. Concordant with these findings were that guards and forwards had a 77.44% and 75.88% proportion of their injuries attributable to soft-tissue injuries, respectively, compared with centers (67.91%) (*P* < .001). Several studies analyzing player position and demographics have not found position to have substantial impact on injury patterns. For example, in the study by Drakos et al.,^27^ the authors found in a 17-year period that player demographics, including height and weight, did not correlate to injury rate. The differences identified in proportion of injuries by position are likely attributed to the different demands of each position, with guards having a greater potential for soft-tissue and lower-extremity injuries due to higher pivoting and cutting rates. Some studies have demonstrated variable return to play rates by position depending on injury. For example, centers may have lower return after lumbar disc herniation^28^ and guards may have lower return after anterior cruciate ligament reconstruction.^29^

### Limitations

Several limitations exist in this retrospective analysis. First, despite the authors efforts for a comprehensive search, due to the online nature of the data collection it is possible that we have missed, or not accurately captured injuries included in the epidemiologic analysis, or identified players diagnosed with COVID-19 in the time frame studied. The epidemiologic data were classified based on public information, and additional details not available may lead to improper categorization of data. There have been numerous strains of the SARS-CoV-2 virus, and the study cannot account for potential differing effects of strains. Furthermore, the advent of vaccines, boosters, and medications to reduce the effects of COVID-19 infection may change the way data is interpreted. For players in the NBA Bubble, additional confounding factors exist contributing to injury rates and player performance. For example, prior data suggests travel to different time zones and circadian rhythm disruptions from travel impact sleep, recovery, and performance.^5^, ^6^, ^7^, ^8^^,^^30^ In addition, the impact of the unique Bubble environment, including but not limited to staying away from players’ homes for a prolonged period, limited interaction with friends and family, and overall stresses from frequent testing and the pandemic are difficult to account for.

## Conclusions

In the NBA seasons from 2016 to 2021, most injuries were to the lower extremity. The SARS-CoV-2 pandemic did not substantially impact injury patterns in the NBA, including locations of injury and type of injury (bony or soft tissue). Furthermore, infection with SARS-CoV-2 does not appear to have a significant impact on performance in basketball-specific or speed and distance measures.

## Disclosure

The authors declare the following financial interests/personal relationships which may be considered as potential competing interests: N.K.P. reports consultant for OrthoPediatrics and educational support from Evolution Surgical, Inc. All other authors (Sachin A., A.R.G., N.C., N.T., A.P.G., J.C.M., Sameer A.) declare that they have no known competing financial interests or personal relationships that could have appeared to influence the work reported in this paper. Full ICMJE author disclosure forms are available for this article online, as supplementary material.

## Supplementary Data

ICMJE author disclosure forms
