# Supplementary material for: Severe Acute Respiratory Syndrome Coronavirus 2 Did Not Substantially Impact Injury Patterns or Performance of Players in the National Basketball Association From 2016 to 2021
Source: Arthrosc Sports Med Rehabil. 2023 Dec 21;6(1):100841. doi: 10.1016/j.asmr.2023.100841 (PMC10776416; doi:10.1016/j.asmr.2023.100841)
Supplement: Supplemental Tables [file mmc2.docx]

**SUPPLEMENTAL TABLES**

**Supplemental Table 1**. NBA injury characteristics from 2016-2021, by season timing.

| General Regions of Injury by Season Timing | | | | | | | | | | | |
| --- | --- | --- | --- | --- | --- | --- | --- | --- | --- | --- | --- |
|  | Total | | Offseason | | Postseason | | Preseason | | Season | |  |
|  | Number | Percentage | Number | Percentage | Number | Percentage | Number | Percentage | Number | Percentage | *p-value |
| All | 3,040 | 100.00% | 24 | 0.79% | 75 | 2.47% | 98 | 3.22% | 2,843 | 93.52% | - |
| Lower Extremity | 1,880 | 61.84% | 21 | 87.50% | 52 | 69.33% | 67 | 68.37% | 1,740 | 61.20% | 0.013 |
| Upper Extremity | 333 | 10.95% | 3 | 12.50% | 10 | 13.33% | 14 | 14.29% | 306 | 10.76% | 0.634 |
| Spine/Core | 230 | 7.57% | 0 | 0.00% | 8 | 10.67% | 7 | 7.14% | 215 | 7.56% | 0.388 |
| **Other** | **597** | **19.64%** | **0** | **0.00%** | **5** | **6.67%** | **10** | **10.20%** | **582** | **20.47%** | **<0.001** |
| Specific Regions of Injury by Season Timing | | | | | | | | | | |  |
|  | Total | | Offseason | | Postseason | | Preseason | | Season | |  |
|  | Number | Percentage | Number | Percentage | Number | Percentage | Number | Percentage | Number | Percentage | *p-value |
| Head | 120 | 3.95% | 0 | 0.00% | 2 | 2.67% | 6 | 6.12% | 112 | 3.94% | 0.469 |
| Neck | 26 | 0.86% | 0 | 0.00% | 1 | 1.33% | 1 | 1.02% | 24 | 0.84% | 0.931 |
| Chest | 13 | 0.43% | 0 | 0.00% | 1 | 1.33% | 2 | 2.04% | 10 | 0.35% | 0.048 |
| Shoulder | 102 | 3.36% | 0 | 0.00% | 4 | 5.33% | 5 | 5.10% | 93 | 3.27% | 0.436 |
| Elbow/Forearm | 38 | 1.25% | 0 | 0.00% | 2 | 2.67% | 3 | 3.06% | 33 | 1.16% | 0.230 |
| Hand/Wrist | 185 | 6.09% | 2 | 8.33% | 3 | 4.00% | 6 | 6.12% | 174 | 6.12% | 0.852 |
| Abdomen | 19 | 0.63% | 0 | 0.00% | 2 | 2.67% | 1 | 1.02% | 16 | 0.56% | 0.132 |
| Groin | 71 | 2.34% | 2 | 8.33% | 1 | 1.33% | 4 | 4.08% | 64 | 2.25% | 0.138 |
| Hamstring | 124 | 4.08% | 0 | 0.00% | 4 | 5.33% | 4 | 4.08% | 116 | 4.08% | 0.724 |
| Hip | 118 | 3.88% | 1 | 4.17% | 1 | 1.33% | 2 | 2.04% | 114 | 4.01% | 0.132 |
| Knee | 556 | 18.29% | 9 | 37.50% | 13 | 17.33% | 19 | 19.39% | 515 | 18.11% | 0.138 |
| Lower Back | 172 | 5.66% | 0 | 0.00% | 4 | 5.33% | 3 | 3.06% | 165 | 5.80% | 0.724 |
| Foot/Ankle | 703 | 23.13% | 7 | 29.17% | 24 | 32.00% | 25 | 25.51% | 647 | 22.76% | 0.508 |
| Infection | 298 | 9.80% | 0 | 0.00% | 3 | 4.00% | 3 | 3.06% | 292 | 10.27% | 0.106 |
| Miscellaneous | 179 | 5.89% | 0 | 0.00% | 0 | 0.00% | 1 | 1.02% | 178 | 6.26% | 0.423 |
| Other Lower Extremity | 308 | 10.13% | 2 | 8.33% | 9 | 12.00% | 13 | 13.27% | 284 | 9.99% | 0.227 |
| Other Upper Extremity | 8 | 0.26% | 1 | 4.17% | 1 | 1.33% | 0 | 0.00% | 6 | 0.21% | 0.011 |
| Injury Types by Season Timing | | | | | | | | | | | |
|  | Total | | Offseason | | Postseason | | Preseason | | Season | |  |
|  | Number | Percentage | Number | Percentage | Number | Percentage | Number | Percentage | Number | Percentage | *p-value |
| Soft Tissue | 2,298 | 75.59% | 16 | 66.67% | 66 | 88.00% | 80 | 81.63% | 2,136 | 75.13% | 0.023 |
| **Bony** | **160** | **5.26%** | **8** | **33.33%** | **4** | **5.33%** | **11** | **11.22%** | **137** | **4.82%** | **<0.001** |
| **Health & Safety/COVID and Miscellaneous** | **582** | **19.14%** | **0** | **0.00%** | **5** | **6.67%** | **7** | **7.14%** | **570** | **20.05%** | **<0.001** |

*Bold *P* values significant at *P*< .002 (Bonferroni correction).

**Supplemental Table 2**. NBA injury characteristics from 2016-2021, by player position.

| General Regions of Injury by Player Position | | | | | | | | | |
| --- | --- | --- | --- | --- | --- | --- | --- | --- | --- |
|  | Total | | Center | | Forward | | Guard | |  |
|  | Number | Percentage | Number | Percentage | Number | Percentage | Number | Percentage | *p-value |
| All | 3040 | 100.00% | 402 | 13.22% | 1140 | 37.50% | 1498 | 49.28% | - |
| Lower Extremity | 1,880 | 61.84% | 223 | 55.47% | 725 | 63.60% | 932 | 62.22% | 0.007 |
| Upper Extremity | 333 | 10.95% | 47 | 11.69% | 100 | 8.77% | 186 | 12.42% | 0.008 |
| Spine/Core | 230 | 7.57% | 33 | 8.21% | 91 | 7.98% | 106 | 7.08% | 0.693 |
| Other | 597 | 19.64% | 99 | 24.63% | 224 | 19.65% | 274 | 18.29% | 0.006 |
| Specific Regions of Injury by Player Position | | | | | | | | |  |
|  | Total | | Center | | Forward | | Guard | |  |
|  | Number | Percentage | Number | Percentage | Number | Percentage | Number | Percentage | *p-value |
| Head | 120 | 3.95% | 22 | 5.47% | 52 | 4.56% | 46 | 3.07% | 0.036 |
| Neck | 26 | 0.86% | 4 | 1.00% | 8 | 0.70% | 14 | 0.93% | 0.771 |
| Chest | 13 | 0.43% | 4 | 1.00% | 4 | 0.35% | 5 | 0.33% | 0.173 |
| Shoulder | 102 | 3.36% | 18 | 4.48% | 25 | 2.19% | 59 | 3.94% | 0.019 |
| Elbow/Forearm | 38 | 1.25% | 1 | 0.25% | 12 | 1.05% | 25 | 1.67% | 0.056 |
| Hand/Wrist | 185 | 6.09% | 28 | 6.97% | 59 | 5.18% | 98 | 6.54% | 0.254 |
| Abdomen | 19 | 0.63% | 1 | 0.25% | 8 | 0.70% | 10 | 0.67% | 0.586 |
| **Groin** | **71** | **2.34%** | **1** | **0.25%** | **21** | **1.84%** | **49** | **3.27%** | **0.001** |
| **Hamstring** | **124** | **4.08%** | **10** | **2.49%** | **21** | **1.84%** | **93** | **6.21%** | **<0.001** |
| Hip | 118 | 3.88% | 14 | 3.48% | 53 | 4.65% | 51 | 3.40% | 0.236 |
| Knee | 556 | 18.29% | 66 | 16.42% | 235 | 20.61% | 255 | 17.02% | 0.036 |
| Lower Back | 172 | 5.66% | 24 | 5.97% | 71 | 6.23% | 77 | 5.14% | 0.468 |
| Foot/Ankle | 703 | 23.13% | 92 | 22.89% | 293 | 25.70% | 318 | 21.23% | 0.026 |
| Infection | 298 | 9.80% | 40 | 9.95% | 121 | 10.61% | 137 | 9.15% | 0.452 |
| **Miscellaneous** | **179** | **5.89%** | **37** | **9.20%** | **51** | **4.47%** | **91** | **6.07%** | **0.002** |
| Other Lower Extremity | 308 | 10.13% | 40 | 9.95% | 101 | 8.86% | 167 | 11.15% | 0.154 |
| Other Upper Extremity | 8 | 0.26% | 0 | 0.00% | 5 | 0.44% | 3 | 0.20% | 0.269 |
| Injury Types by Player Position | | | | | | | | |  |
|  | Total | | Center | | Forward | | Guard | |  |
|  | Number | Percentage | Number | Percentage | Number | Percentage | Number | Percentage | *p-value |
| **Soft Tissue** | **2,298** | **75.59%** | **273** | **67.91%** | **865** | **75.88%** | **1,160** | **77.44%** | **<0.001** |
| Bony | 160 | 5.26% | 35 | 8.71% | 53 | 4.65% | 72 | 4.81% | 0.004 |
| Health & Safety/COVID and Miscellaneous | 582 | 19.14% | 94 | 23.38% | 222 | 19.47% | 266 | 17.76% | 0.037 |

*Bold *P* values significant at *P*< .002 (Bonferroni correction).
